# Does tranexamic acid reliably reduce blood loss in proximal femur fracture surgery?

**DOI:** 10.1007/s00068-022-02042-6

**Published:** 2022-07-19

**Authors:** A. Fenwick, I. Antonovska, M. Pfann, J. Mayr, A. Wiedl, S. Nuber, S. Förch, E. Mayr

**Affiliations:** Department of Trauma, Orthopedic, Plastic and Hand Surgery, University Hospital of Augsburg, Stenglinstrasse 2, 86156 Augsburg, Germany

**Keywords:** Blood loss, Proximal femur fracture, Transfusion rate, Tranexamic acid

## Abstract

**Purpose:**

The aim of our study was to investigate the use of tranexamic acid in patients with proximal femoral fractures and compare the total blood loss, transfusion rates, complications, and the application method.

**Methods:**

A retrospective single center cohort study (level I trauma center) with 1479 patients treated operatively for a proximal femoral fracture between January 2016 and June 2020 was performed. 1 g of tranexamic acid was applied (systemic, topic or combined application). Patient data, surgical procedure, complications, and mortality were assessed. Hemoglobin levels, blood loss and transfusion rates for patients with and without tranexamic acid and the application methods were compared.

**Results:**

667 femoral neck fractures, 701 pertrochanteric and 109 subtrochanteric fractures were included. Mean age was 80.8 years. 274 patients received tranexamic acid. At admission average hemoglobin was 12.2 g/l. Hemoglobin drop postoperatively was less after tranexamic acid (9.72 vs. 9.35 g/dl). Transfusion rates were lowered significantly by 17.1% after tranexamic acid. Blood loss was reduced for all patients after tranexamic acid independent of fracture morphology. The combination of 1 g i.v. and 1 g topical-applied tranexamic acid seems to be more effective. Complication rates did not differ.

**Conclusion:**

Tranexamic acid is effective in reducing blood loss and transfusion rates, without increasing the risk of thromboembolic events after proximal femoral fractures. For open reduction and nailing and arthroplasty in fracture setting combined topical and single i.v. application seems most effective and closed reduction with nailing can be treated by single dose i.v. application of 1 g tranexamic acid.

## Purpose

Hip fractures are common and increasing [1, 2]. In Germany the incidence of proximal femur fractures has risen by 24% from 2009 to 2019 [3]. Surgery performed depending on fracture morphology is total- or hemi arthroplasty, intramedullary nailing or dynamic hip screw fixation and mortality rates are as high as 30% during the first postoperative year [4]. Impairment of quality of life, daily activities and postoperative mobility is of vital importance especially to the mainly affected and growing geriatric population. The total amount of blood loss due to trauma itself and surgery resulting in anemia plays a crucial role in postoperative outcome and can prolong recovery [5–9]. Postoperative anemia and hypovolemia can worsen cardiac or renal symptoms in patients suffering from cardiac preconditions or renal dysfunction [10, 11]. Geriatric patients are especially vulnerable to these blood loss- associated problems. A rising number of patients with additional anticoagulants pose a further risk of an even higher blood loss.

Tranexamic acid is a competitive binder to the lysin- binding site of plasminogen and inhibits transformation into plasmin and therefore reduces fibrinolysis and stabilizes existing blood clots [12]. It was deemed to reduce blood loss and reduce transfusion rates. Trauma studies carried out in the US army showed reduced mortality rates after introduction of tranexamic acid in comparison to the control groups [13]. By now it has found its use in elective hip and knee arthroplasty as well as spine surgery and polytraumatized patients and has shown to be an efficient and cost saving method to reduce transfusion rates and speed up postoperative recovery [14–16]. The currently unsolved problem of possible vascular adverse events, especially deep vein thrombosis, remains [17]. Patients with hip fractures are particularly vulnerable and at risk for deep vein thrombosis so special consideration should be given to these possible complications that might restrict the use of tranexamic acid. Recent studies have been investigating the use in mono- trauma patients concentrating on femoral neck fractures [18–21].

Whilst former studies have concentrated on restricted patient groups or elective settings, the prime aim and novel aspect of our study was to involve all patients with proximal femoral fractures including per-, subtrochanteric-, and femoral neck fractures and therefore investigate the benefit of tranexamic acid on total blood loss, transfusion rates and complications depending on fracture morphology and subsequent surgery and further differentiate between the application methods.

## Methods

### Data acquisition

We performed a retrospective cohort single center study (level I trauma center), level III evidence, coherent with the STROBE statement, including all patients treated operatively for a proximal femoral fracture between January 2016 and June 2020 [22]. Femoral neck, pertrochanteric and subtrochanteric fractures were included. We excluded greater trochanteric fractures, periprosthetic fractures as well as referrals for revision surgery and polytraumatized patients to avoid bias for other blood loss reasons. Patients without pre- or postoperative labs, with concomitant fractures and patients undergoing further surgical procedures during the first 6 days after admission for proximal femoral fracture were excluded for blood loss calculation to avoid false conclusions.

The study conducted was approved by the local Ethics Committee of the University of Regensburg and fulfills the standards of the declaration of Helsinki (ID: 20-2155-101).

The charts were reviewed for demographic data: age, gender, body mass index BMI, Charlson Comorbidity Index CCI [23] and ASA classification [24], fracture morphology, medication, complications (deep infection, embolism, stroke, seizure, myocardial infarction), revisions, labs and blood transfusions. Special attention was paid to thromboembolic events which could be due to tranexamic acid. Patients admitted again with a fracture on the contralateral side during the reviewed period were included again as a separate case.

### Therapy

Minimal invasive intramedullary nailing PFNa (proximal femur nail antirotation, Fa. Synthes) was performed for pertrochanteric fractures and lateral femoral neck fractures. Subtrochanteric fractures were addressed by open reduction, cerclage and intramedullary nailing in side- positioning. Dependent on pre- operative mobility and comorbidities as well as fracture morphology total or hemi arthroplasty (cemented or uncemented) was performed for medial femoral neck fractures. Patients without anticoagulants were treated within 24 h. For patients on direct anticoagulants (DOACs) the last intake was recorded. Patients with renal clearance > 50 ml/ml were treated within 24–48 h and patients with GFR (glomerular filtration rate) < 50 ml/min after 48 h of the last intake of DOAC according to our in- house protocol. Postoperatively venous thromboembolism prophylaxis was given from day one with Enoxaparin 40 mg subcutaneously to patients without anticoagulants. Anti-platelet therapy was continued. DOACs and Warfarin were substituted with Tinzaparin-sodium according to patient weight. Warfarin was reversed with Vit K if possible, preoperatively until Quick was > 60%. No prothrombin complex concentrate (PPSB) was given. Neither DOACs nor Warfarin were bridged. Mobilization was initiated from day one on after surgery with full weight bearing for all patients.

The blood loss was calculated using the Mercuriali formula [25], which is based on pre- and postoperative hematocrit and the number of transfused RBCs (Red blood cell) as well as patients` blood volume. This is calculated by the Nadler formula [26], which is a specific calculation according to gender and height.

Women: BV (l) = height (m)3_0.3561 + weight (kg)_0.03308 + 0.1833 [26].

Men: BV(l) = height (m)3_03669 + weight (kg)_0.03219 + 0.6041 [26].

Estimated blood loss: BV × (Hct_preop_–Hct_day 5 postoperative_) + ml of transfused RBC [25].

Transfusion protocol implemented that hemoglobin levels under 7 g/dl received blood transfusions if consented. Between 7 and 8 g/dl transfusions were done depending on symptoms and cardiovascular risk factors.

### Tranexamic acid

Tranexamic acid protocols were introduced mid 2018. All patients before 2018 form a large control group without tranexamic acid as all surgical protocols were checked for administration of tranexamic acid. 1 g Tranexamic acid was administered in the operating room (OR) if patient was viable, either systemically or directly into the surgical site at the end of the procedure before closing up or combined locally and intravenously. If tranexamic acid was applied into the surgical site, no drains were inserted. As tranexamic acid for proximal femur fractures is still off- label use strict exclusion criteria were introduced consisting of the known contraindications even if this may lead to a bias: pulmonary disease including pulmonary hypertension, myocardial infarction, deep vein thrombosis or coagulopathy, stroke or pulmonary embolism in patient history or a high thromboembolic risk.

### Statistical analysis

Statistical analysis was carried out with IBM SPSS Statistics (version 27; IBM Deutschland Ltd., Ehningen, Germany). Normal distribution of all data was verified (Shapiro wilk test). The student’s *t* test, chi square, ANOVA variance and binary logistic regression were used to determine differences and influencing factors regarding complications and mortality; 95% confidence intervals and standard deviations were calculated. For data without normal distribution the Wilcoxon Rank Test was used. The significance level was set at 5% (*α* = 0.05).

## Results

### Demographic data

1479 patients were included in the investigation. 68.9% were female and 31.1% male with an average age of 80.8 years (range 18–103; SD 10.8). The mean BMI was 24.44 kg/m^2^ (range 13.5–66.4; 11.7–66 kg/m^2^). The cohort consisted of 677 femoral neck fractures, 701 pertrochanteric fractures and 109 subtrochanteric fractures. In 335 cases total hip endoprothesis was implanted. 342 patients received a hemiarthroplasty. Intramedullary nailing was done in 810 cases. Between the fracture types there were no statistical differences of gender or BMI distribution. Patients with tranexamic acid retrospectively had slightly less comorbidities especially the group with systemically administered tranexamic acid (Table 1). Both patients with and without tranexamic acid were mainly classified as ASA II and III (tranexamic acid 87.6%, without 88.5%). Of all patients 62.9% could be treated within 24 h and another 25.8% met the 48-h time limit. The average waiting time for surgery was 25.9 h (range 0.95–140.8; SD 20.2 h) after hospital admission. There was no difference in the average length of hospital stay which for both groups of patients was 14 days.

**Table 1:** Patient characteristics. Number of patients with percentage or as mean

	No tranaxamic acid (%)	Tranexamic acid intravenousyl (%)	Tranexamic acid i.v. + locally (%)	Tranexamic acid locally (%)	*p* value
Number of patients (with labs)	1205	155	59	10	
BMI (kg/m^2^)	24.44	24.48	23.85	25.8	*p* < 0.587
Age (years)	81.28	78.53	77.95	82.7	*p* < 0.04
Charlson comorbidity index (points)	5.94	5.19	5.97	7.36	*p* < 0.02
Complications					
Internal bleed	12 (0.99)	1 (0.64)	1 (1.69)	None	*p* < 0.797
Pulmonary embolism	9 (0.75)	1 (0.64)	–		
Deep vein thrombosis	6 (0.5)	–	–		
Myocardial infarction	9 (0.75)	–	–		
Stroke	6 (0.5)	1 (0.64)	1 (.69)		
Seizure	4 (0.33)	–	–		
Wound infection	28 (2.3)	7 (4.5)	3 (5)		
Mortality	41 (3.4)	–	3 (5.08)		
Blood loss in ml	1435.34	1225.9	1139.55	895.86	*p* < 0.000
Hemoglobin preoperative	12.2	12.9	12.6	12.8	
In g/dl					
Number of patients receiving RBC transfusion	373 (30.9)	32 (20.6)	6 (10.1)	0	*p* < 0.021
RBC units transfused	717	56	20	0	

### Tranexamic acid

**Fig. 1:**
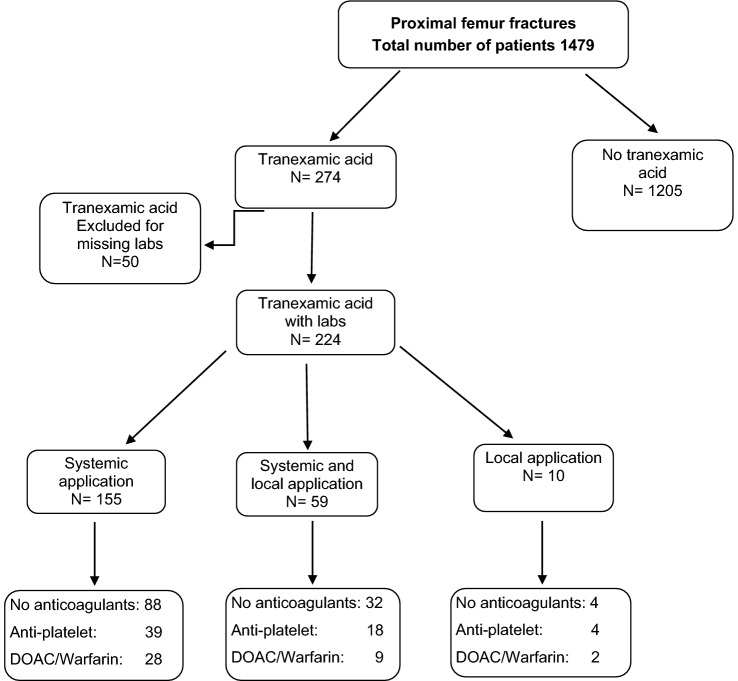
Flowchart patient inclusion criteria and group distribution according to anticoagulants

274 patients (14.18%) received tranexamic acid intraoperatively, of these, labs were available for 224 patients. 185 of the latter were treated with a total or hemi arthroplasty. A further 39 patients received tranexamic acid while intramedullary nailing (Fig. 1). Of these 20 received open reduction plus cerclage. In these cases, tranexamic acid was always given intravenously, whilst a third of the arthroplasties also had locally administered tranexamic acid.

### Blood loss

**Fig. 2:**
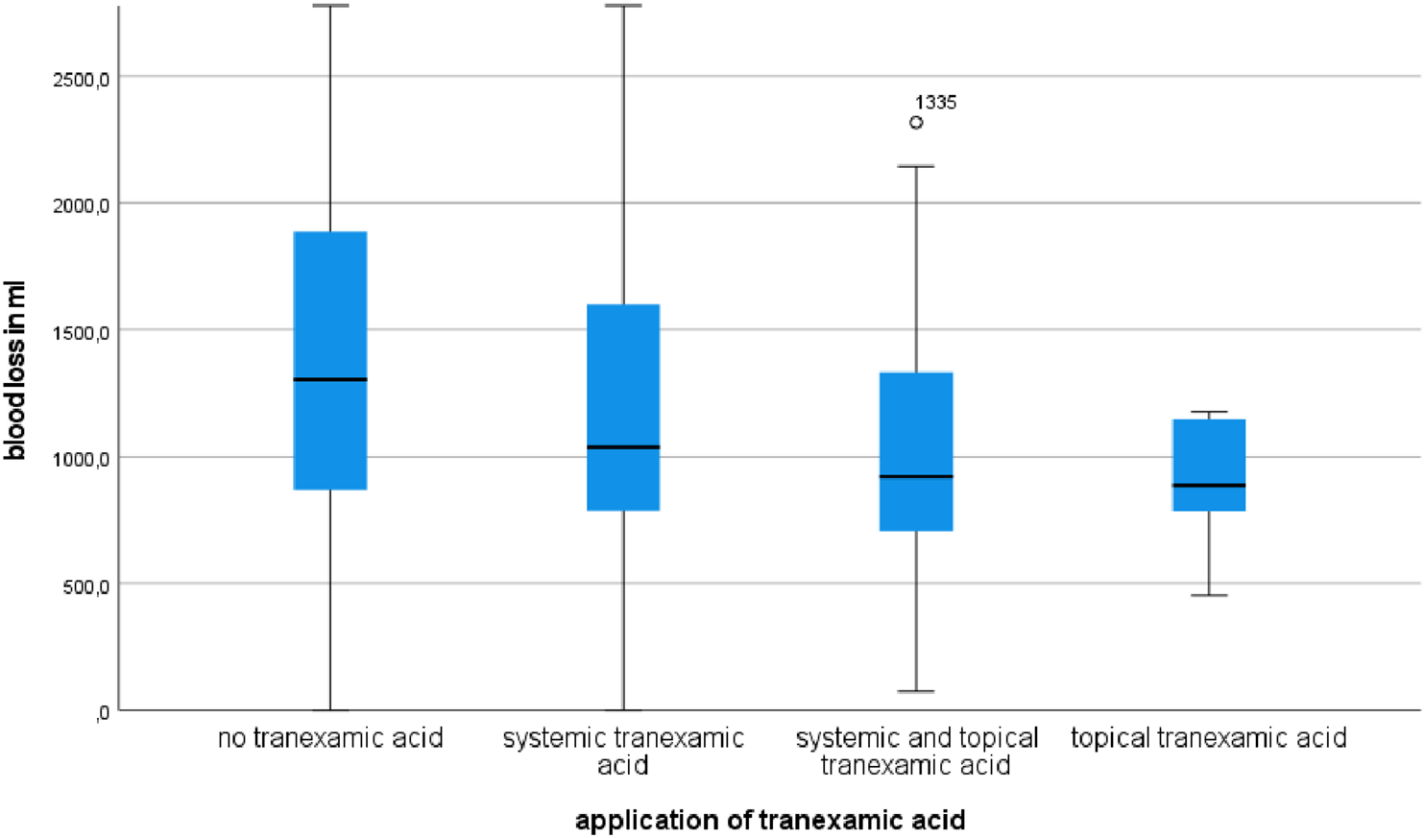
Comparison of total blood loss after different application methods of tranexamic acid to patients without tranexamic acid

On average patients without tranexamic acid had a blood loss of 1435.34 ml (range 247.3–8868.9; SD 840.6 ml). In 156 cases tranexamic acid was administered systemically. In this case blood loss was significantly lower (1225.9 ml, range 172.8–7387.9; SD 733.46 ml). Combining intravenous and application directly into the wound further reduced the total blood loss (1139.55 ml; range 73.4–4107.6; SD 810.18 ml). The lowest blood loss was registered for patients who only received tranexamic acid directly into the wound during surgical procedure (895.86 ml; range: 453.5–1176.2; SD 237.78 ml), *p* < 0.000, Fig. 2.

A further investigation into the different surgical procedures performed and therefore dividing the patients into 3 groups (arthroplasty, minimal invasive nailing and open reduction and nailing + cerclage) reveals overall lower blood loss for administration of tranexamic acid (Table2). Arthroplasty showed a reduction of blood loss by 14.4% as against without systemic tranexamic acid (1128.34 vs. 1319.46 ml, Δ 191.12 ml) and open reduction and nailing also showed a decrease in blood loss (1768.29 vs. 1905.54 ml, Δ 137.25 ml; 7.2%) as well as minimally invasive nailing during which blood loss was reduced by 12.5% (1290.69 vs. 1476.63 ml, Δ 185.94 ml).

**Table 2:** Subgroup analysis of surgical procedures, their demographic data and the effects of tranexamic acid on blood loss

Parameter (including range and SD)	Arthroplasty	Arthroplasty + tranexamic acid	Closed reduction and nailing	Closed reduction and nailing + tranexamic acid	Open reduction and nailing	Open reduction and nailing + tranexamic acid	*p* value
Number of patients (with labs)	467	185	659	19	79	20	
BMI	24.2 (13.5–44.4; SD 4.2)	24.3 (14.5–44; SD 4.6)	24.3 (14–0-66.2; SD 4.7)	26.1 (14.7–37.1; SD 6.4)	26.6 (15.2–42.9; SD 4.9)	25.38 (19.5–32.5; SD 3.7)	0.203
Age	78.8 (23–103)	77.9 (41–96)	81.3 (25–103)	78.6 (64–91)	78.6 (18–98)	77.6 (37–95)	0.957
Charlson Comorbidity Index	5.81 (0–15; SD 2.6)	5.48(1–15; SD2.4)	6.09 (0–15; SD 2.4)	5.94 (2–9; SD 1.7)	5.42 (0–11; SD 2.4)	5.04 (0–11; SD 2.7)	0.001
Blood loss im ml	1319.4 (435.8–6735.6; SD 760.1)	1128.34 (172.8–4107.6; SD 710.5)	1476.63 (262.6–5338.4; SD 831.6)	1290.69 (73.4–7387.9; SD 1847.9)	1905.54 (247.3–8868.9¸ SD 1115.0)	1768.29 ml (787.3–3245.8; SD 681.1)	0.000

### Transfusion rates

In overall 799 RBCs were transfused of which 723 were needed in the group without tranexamic acid. 373 (30.9%) patients received transfusions without having had tranexamic acid while only 38 patients (13.8%) were given RBCs in the group after intraoperative tranexamic acid independent of application method. This is a significant drop of RBC transfusions after proximal femoral fractures (*p* < 0.021). Preoperative hemoglobin did not differ significantly (12.20 vs. 12.85 g/dl). Postoperative hemoglobin was significantly higher for patients with tranexamic acid (9.72 SD14.9 vs. 9.35 g/dl SD14.76; hematocrit in %: 21.1 SD12.3 vs. 18.7 SD12.3).

### Anticoagulants

Slightly more than 50% of the cohort had no rheological therapy preoperatively. 17.9% of the cohort were on anticoagulants at admission. Of these patients 222 were on DOACs (Apixaban, Rivaroxaban, Edoxaban), 118 on Warfarin. A further 585 patients had anti-platelet therapy (ASS or Clopidogrel). Of all the patients receiving tranexamic acid there were patients on anticoagulants in all groups (Fig. 1). The blood loss for patients on anticoagulants (1372.1 ml; 163.6–8868.9; SD 1009.26 ml) was lower than the average blood loss without tranexamic acid but no significant difference between the amongst the groups was seen, *p* = 0.139.

### Complications

The total complication rate was 21.9% (surgical site infection, urinary tract infection, pneumonia, pulmonary embolism, thrombosis, dislocation, fracture). There was no significant difference between the complication rates for patients with and without tranexamic acid (18.6 vs. 22.2%). Thromboembolic complications, especially pulmonary embolism or stroke, or STEMI did not occur more often after administration of tranexamic acid (Table 1). No complications occurred in the group of locally given tranexamic acid. There were no deaths in the intravenous and topical group and 3 patients died in the combined intravenous/topical group. Overall, there was no significant change in mortality rates after tranexamic acid (*p* < 0.323).

## Discussion

There is some hesitation about standard use of tranexamic acid, especially in case of trauma with hip fractures, as these are connected to a significant high risk themselves for thromboembolic events [27, 28].

The aim of our study was to evaluate the use of tranexamic acid in trauma patients including the main types of proximal femur fractures and compare the application methods. The findings of our study support the use of tranexamic acid to reduce the amount of blood loss after proximal femur fractures without increasing complication rates or mortality.

Randomized control studies have shown tranexamic acid to be an effective way to reduce postoperative blood loss and simultaneously reduce the amount of required blood transfusions [29]. Zufferey et al. [20] proved the necessity of a transfusion to drop by 30% and Tengberg et al. [21] showed a significant decrease of total blood loss in a similar setup with extracapsular hip fractures. Both claimed an effective reduction of the above mentioned but also recorded different problems. Whilst Zufferey recorded an increase of vascular events, mainly asymptomatic deep vein thromboses, Tengberg stated a higher 90 days mortality rate (27.2 vs 10%) [20, 21]. Further studies also underline our findings of both reduced blood loss and decreased transfusion rates and have postulated that tranexamic acid should be a standard protocol for proximal femoral fractures [18, 19, 30]. Our findings support that subtrochanteric fractures lead to the highest total blood loss and can also benefit from the use of tranexamic acid, as also proven by Lei and Tengberg [21, 31].

Controversary remains about the accompanying risks and complications. For elective surgery a careful patient selection is possible in contrast to a trauma situation with immediate intervention necessary. Furthermore, hip fractures themselves are already associated with a high thromboembolic risk. However, we did not see an increase in complication rates. This is supported by Xie et al. [32] who also recorded comparable general complications rates and Geddes et al. [27] who specifically recorded thromboembolic events and could not see an increase after tranexamic acid. An extensive meta-analysis was performed and with the exemption of a minority of studies they concluded that tranexamic acid did not pose a higher risk for thromboembolic events [33]. We did not record an increase of complication rates, especially thromboembolic complications. Thus, in the group of tranexamic acid injected directly into the surgical site there were no complications recorded. This might be due, however, to the very small number of patients.

Numerous studies have now been conducted with some inconsistency about methods of application and dosage as well as timing of tranexamic acid. Two meta-analysis including knee and hip arthroplasty compared application methods as well as dosage and timing of dosage and concluded that intraarticular and single i.v. application of tranexamic acid had similarly effective results in reducing total blood loss, transfusion rates and postoperative hemoglobin drop [34, 35]. Interestingly, repeat doses of tranexamic acid led to a higher blood loss and higher drop of hemoglobin. Sun also concluded that the combined application route showed no increase in thromboembolic events [34].

Abdallah et al. again showed in a trial of patients with knee arthroplasty that the best application method seemed to be a combined approach of i.v. and local tranexamic acid as it led to a significant drop of blood loss and a reduced transfusion rate [36]. Similar results could also be reproduced for knee- and hip arthroplasty showing the combined application to be superior to either topical or intravenous application [37, 38]. Our findings coincide with the above mentioned and support a combined use of single i.v. dose (1 g) and locally injected tranexamic acid (1 g) in a trauma setting for proximal femur fractures having even better results than plain intravenous application.

In our cohort the best results were seen for ten patients who received tranexamic acid (1 g) only directly into the surgical site before closing up the fascia during arthroplasty. Tranexamic acid has a biological half time of around three hours and more than 90% is eliminated within the first 24 h. The plasma protein binding is only 3% at therapeutic plasma levels [12]. Even so, we are not able to explain the very much lower blood loss after solely applying tranexamic acid directly into the wound performing arthroplasty. It may only be a bias result of the low number of patients who received this application. Possibly due to trauma leading to the main bleeding the importance of stabilizing existing clots is more important and efficient locally. But this does still not explain local administration being more successful than the combination of topical and systemic administration and leads us to the clear limitations of our study.

The main limitation is the retrospective and unrandomized design of the study. The total number of patients receiving tranexamic acid is limited and is limited even further by subdividing the groups for more detailed analysis. Furthermore, the number of patients with tranexamic acid injected locally into the surgical site is very small and therefore there is a bias about the positive effects on the blood loss seen in this application method in our investigation. Further investigation with higher number of patients should be undertaken.

## Conclusion

Proximal femoral fractures pose a greater risk of significant total blood loss and often require blood transfusions. Severe blood loss is associated with postoperative anemia, prolonged hospital stay and slow recovery. Application of tranexamic acid is an effective way to reduce blood loss and transfusion rates independent of fracture morphology, surgical procedure, or underlying medication such as anticoagulants. After careful pre patient selection it is a low-cost method without a significantly increasing the risk of thromboembolic events after proximal femoral fractures. For open reduction and nailing or arthroplasty in fracture setting combined topical and single i.v. 1 g dose seems most effective and closed reduction with nailing can be treated by single dose i.v. application of 1 g of tranexamic acid.
